# Health Seeking Behaviour and Treatment Intentions of Dengue and Fever: A Household Survey of Children and Adults in Venezuela

**DOI:** 10.1371/journal.pntd.0004237

**Published:** 2015-12-01

**Authors:** Jelte Elsinga, Erley F. Lizarazo, Maria F. Vincenti, Masja Schmidt, Zoraida I. Velasco-Salas, Luzlexis Arias, Ajay Bailey, Adriana Tami

**Affiliations:** 1 Department of Medical Microbiology, University of Groningen, University Medical Center Groningen, Groningen, The Netherlands; 2 Instituto de Investigaciones Biomédicas, Universidad de Carabobo, Maracay, Venezuela; 3 Departamento de Biología, Facultad Experimental de Ciencia y Tecnología, Universidad de Carabobo, Valencia, Venezuela; 4 Population Research Center, Faculty of Spatial Sciences, University of Groningen, Groningen, The Netherlands; 5 Departamento de Parasitología, Facultad de Ciencias de la Salud, Universidad de Carabobo, Valencia, Venezuela; Makerere University, UGANDA

## Abstract

**Background:**

Dengue in Venezuela is a major public health problem with an increasing incidence of severe cases. Early diagnosis and timely treatment influences the outcome of dengue illness, as delay in care-seeking is significantly associated with complications leading to severe dengue. We aimed to understand patterns of health seeking behaviour (HSB) in individuals exposed to high dengue incidence in order to improve early attendance to health centres.

**Methods:**

Between September 2013 and February 2014 a cross-sectional household survey was performed in Maracay, Venezuela. Intended HSB of adults and children’s parents/guardians was assessed with respect to fever or suspected dengue. Data was collected through structured questionnaires from 105 individuals.

**Results:**

Most individuals felt at risk of dengue and believed it could be a deadly disease. In the case of suspected dengue, the majority (60%) would choose to first seek medical help versus first treating at home, in contrast to 11% in the case of fever. Amongst those who decided to visit a doctor, a suspected dengue infection would prompt them to search medical help earlier than if having only fever (p<0.001). Multivariate analysis modelling showed that the independent factors associated with the intention to firstly visit a doctor versus treating at home in the case of dengue were feeling at risk (OR = 3.29; p = 0.042) and being an adult (as opposed to caring for a child as a parent/guardian; OR = 3.33, p = 0.021), while having had a previous dengue infection (OR = 0.29; p = 0.031) and living in the neighbourhood Caña de Azúcar (OR = 0.28, p = 0.038) were negatively associated with seeking medical care as their first action.

**Conclusion:**

Knowledge of HSB related to dengue is scarce in the Americas, our study attempts to contribute to a better understanding of HSB in this region. Improving early dengue disease recognition and awareness may enhance prompt attendance to medical care in affected populations and thereby reduce mortality and severity of dengue. Especially for those with a previous dengue infection, efforts have to be made to promote prompt health centre attendance.

## Author Summary

The rapid spread of dengue in the last decade has brought this disease higher in the public health agenda worldwide. Dengue is transmitted by day-biting mosquitoes and typically presents with sudden fever and flu-like symptoms. In a proportion of cases, severe disease can ensue which if not promptly treated can be lethal. In Venezuela, the frequency of dengue epidemics and severe disease is on the increase. Delay in care-seeking has been related to the development of serious disease complications. In this study, we describe the health-related behaviour intentions people have when they face a dengue infection, to find ways to promote prompt medical care attendance. Few studies of this kind exist in the Americas. We found that in case of fever alone, people would mostly prefer to first treat the disease at home while in the case of suspected dengue they would choose to promptly visit a doctor. These intentions are encouraging. However, since people often do not realize they have dengue, they may not perform their planned behaviour. Moreover, we found several factors that made people choose to visit promptly a doctor: being an adult (as opposed to caring for a child as a parent/guardian), place of residence, feeling at risk and not having had a previous dengue infection. Therefore, we suggest that raising risk perception and the knowledge on dengue symptoms and transmission route could improve earlier health centre attendance lowering the risk of clinical complications leading to severe dengue and death.

## Introduction

Dengue fever, a viral vector-borne disease spread by the day-biting mosquitoes *Aedes aegypti* and *A*. *albopictus*, is a global health problem of increasing importance [1]. Currently, dengue affects over 2.5 billion people living in dengue endemic areas, which comprises 40% of the world’s population [2]. According to estimations of the WHO, 50–100 million dengue infections occur every year, leading to 500 000 cases of severe disease that need hospitalisation [2]. However, recent estimations speak of approximately 400 million dengue infections annually [3]. Where in the 1950s dengue cases were reported in only nine countries, today more than 125 countries in the tropics and subtropics are endemic for dengue [4]. In the Americas, almost all countries struggle with recurrent epidemics [5]. The poverty, poor sanitation and overcrowding that accompanies the uncontrolled urbanisation in this region creates environments in favour of vector-breeding and rapid spread of the virus, which leads to serious obstacles in disease control [6].

Dengue has become a major public health problem in Venezuela, with epidemics of increasing magnitude regularly occurring against a background of an established endemic situation. Initial descriptions of dengue-like illness in Venezuela based on clinical manifestations date from 1828 and 1946 [7]. Since the first dengue hemorrhagic fever epidemic reported in the country in 1989–1990 and the second in the Americas [8], the frequency of severe cases has increased. Between 1989 and 2007, the highest proportion (35%) of severe dengue cases within the Americas were reported in Venezuela [9]. In effect, dengue transmission in Venezuela has become perennial with poverty-related socio-economic factors and behavioural determinants fuelling the increasing incidence of dengue in the urban areas of the country [9, 10].The most recent and largest dengue outbreak took place in 2010 with more than 120.000 reported cases, of which 8% represented severe cases [11].

Early diagnosis and adequate supportive care are of great importance in the management of dengue so as to avoid the development of complications and severe disease. Thereby, early treatment intervention can reduce the case fatality rate from 20% to 1% or less [1, 12]. While knowledge and possibilities to diagnose and treat dengue fever increase, efforts have to be made to make these new developments accessible for those who have a dengue infection. An important factor to be taken into consideration is the patient’s health seeking behaviour (HSB), because for early diagnosis and supportive care, people must have the intention and the means to seek medical care early in the disease. Therefore, local studies on health believes and practices, HSB and access to care with respect to dengue fever are needed to identify barriers and opportunities for applying these new developments in diagnostics and treatment [13]. Insights in HSB of dengue could help to attain a reduction of late diagnosis, an increase of treatment adherence and improvement of health promotion strategies applied to a specific culture [14].

Two important health behaviour theories are the Health Believe Model (HBM) [15] and the Theory of Planned Behaviour (ToPB) [16]. A central concept in the HBM is the ‘perceived susceptibility’, which refers to the perceived chance of acquiring a condition (in this article also referred to as ‘risk perception’). The ‘perceived susceptibility’ and the ‘perceived severity’ leads to the formation of ‘perceived threat’ of a certain condition. The likelihood of performing a certain health behaviour is directly linked to a) the perceived threat, b) the perceived benefits and barriers of the suggested behaviour change, c) the self-efficacy and d) the cues to action [15, 17]. The ToPB links the attitude towards behaviour, subjective norms and perceived behavioural control to behavioural intentions and behaviour [16, 18].

In Venezuela, patients with a suspected dengue infection tend to seek medical help beyond the third day after the onset of fever [19]. At this time, the patient may be already critically ill [20]. Delay in care-seeking is found to be significantly associated with complications leading to severe dengue [21], which stresses the importance of understanding HSB and access to care for dengue patients. However, research on HSB applied to dengue appears to be scarce, especially in the Americas, as the majority of studies addressing (partially) this topic have been performed in Asia [22–28]. Moreover, the published literature on HSB related to dengue was not [22–25, 27, 28], or was only partly [26] based on health behaviour theories.

This study aims to understand the patterns of HSB in the Venezuelan population exposed to high dengue incidence in order to find ways to improve early attendance to health centres and medical care. We compared HSB intentions of adults and of parent/guardians with respect to their children in the case of fever or suspected dengue. By using quantitative data supplemented with qualitative data based on the HBM, we aim to present a better insight in social, psychological and cultural motives of the intended behaviour and attitudes.

## Materials and Methods

### Study site

In August 2010 a prospective, community-based cohort study was set up in Maracay, Venezuela. Maracay is one of the largest cities of Venezuela with dengue hyper-endemicity [9, 29]. It is the capital of Aragua state with an estimated of 1.300.000 inhabitants [30]. Aragua state witnessed the highest incidence of dengue in Venezuela in 2012, reaching nearly 7000 reported cases of which 2% were severe [11]. The study site and design has been described elsewhere [10]. Briefly, three neighbourhoods within Maracay called Candelaria, Caña de Azúcar and Cooperativa were selected for their high dengue incidence. All neighbourhoods are served by public (governmental primary and secondary) health centres [11].^.^ Patients that require further specialised treatment are referred to the main public tertiary level hospital, the Hospital Central de Maracay. Private hospitals and clinics are also part of the health system.

### Study design

Within the cohort study, 2014 individuals aged 5–30 years old living in 840 households were enrolled at the start of the study and followed annually [12]. The present study was performed during the September 2013 and February 2014 annual survey. A cross-sectional survey of a sub-sample of the cohort participants was carried out to gather quantitative and qualitative data on HSB intentions at community level of the general population exposed to dengue.

The study was conceptualised to first address the interviewees’ HSB with respect to fever only as a symptom of several possible diseases. Subsequently, individuals were enquired about their HSB in regard to a specific disease, in this case a suspected dengue infection. Therefore, the study uses aspects of the HBM theory to incorporate the concepts of susceptibility and severity in the analysis to understand the HSB after the onset of fever as this symptom could lead to the individual perceiving susceptibility to multiple conditions [15, 17]. This theory was not applied in the data collection but aided in the analysis and interpretation of the results.

### Study population

A randomized sub-sample of approximately 260 households included in the cohort study was selected. One individual was interviewed in each household. The intention was to interview an equal number of adults and parents or guardians of children (<18 years old) who were already participating in the cohort study. Adults (18 years and older) were randomly chosen from all present adults at the moment of visiting the selected households.

### Data collection

A structured questionnaire, the HSB-questionnaire, was developed containing pre-coded and open questions on socio-demographic and socio-economic details, knowledge of dengue symptoms and dengue transmission, risk perception, pathways of HSB in relation to presenting fever and suspicion of dengue infection. With respect to the parents/guardians interviewed, questions on HSB and risk perception referred to the child, while adults were interviewed with respect to their own attitudes and practices. The questionnaires were prepared in English, translated to Spanish, pre-tested and adapted in a pilot study. Data on socio-economic variables were collected from a household questionnaire which was applied as part of the annual survey of the cohort study.

#### Socio-demographic and socio-economic characteristics

From the HSB-questionnaire we gathered demographic characteristics of the interviewed person: age, place of residence (neighbourhood), level of education, occupation and religion. Additional socio-economic data was gathered in the household questionnaire, and included characteristics of the residence (type of residence, number of persons living in the household, number of rooms (bathrooms not included), roof type, floor material, availability of all public services (piped water, electricity, gas and garbage collection), total income per household, and ownership of 19 different household items. Socio-economic data was used as proxy markers to estimate socio-economic status of the individuals.

#### Knowledge on dengue

Individuals were asked if they had heard about dengue previously. Dengue knowledge was assessed through open questions in two aspects: a) transmission knowledge by asking “how do you think people get infected with dengue?” and b) symptoms knowledge with the question “which are the symptoms of dengue?”. Correct answers to the open questions were: ‘by (the bite of) a mosquito’ (transmission knowledge); and ‘fever, headache, eye pain, body pain, face redness/rash, muscle pain, abdominal pain, sore throat, vomiting, diarrhoea, malaise, nausea, bleeding, fainting, dizziness, itching’ (symptoms knowledge). The ‘overall knowledge score’ (maximum score: 17 points) was derived by adding one point per correct answer, which equalled the sum of the transmission (maximum score: 1) and the symptom (maximum score: 16) knowledge scores (S1 Table). This methodology and scoring of knowledge was used in several other studies [31–33].

#### Behavioural characteristics

These were enquired through open questions: ‘what would you do if you/your child had fever’; and ‘what would you do if you think that you/your child have/has dengue’. Pre-coded options were: a) staying and treat at home (‘home treatment’), b) ‘visit a medical doctor’, c) ‘alternative treatment’ (alternative medicine practitioner, traditional healer, community leaders, friends), d)‘call a medical doctor’, e)‘another action’, or f)‘no action’. After each action mentioned, the person was asked: ‘would you do anything else?’ for up to three additional actions. If ‘home treatment’ was mentioned, interviewees were asked to specify the type of home treatment. If ‘go to the doctor’ was mentioned, they were asked when and to which health centre they would choose to go and if they would visit other health centres if needed. After asking if the interviewed individual chose to seek a medical doctor in the case of a possible dengue infection, we enquired what would make him/her decide to seek medical help. Possible pre-coded options were: the number of days of fever, temperature of the fever, the appearance of new symptoms or another reason (which was written down). If any of the first three options was mentioned, respondents were asked to specify the number of days, degree of temperature and type of symptoms.

#### Risk perception

Perception of the risk of acquiring a dengue infection (either referred to children or adults) was assessed during the interview with the following questions: ‘Do you think that you are at risk of contracting dengue?’ or ‘Do you think that your child is at risk of contracting dengue?’. The possible answers were ‘yes’, ‘no’, or ‘I don’t know’. These questions were then used as proxy for measuring perceived susceptibility from the HBM.

#### Qualitative data

During the interview, some individuals spontaneously explained what motivated the answers they gave. In other occasions we asked the individual to explain the reasons for their answer(s). Questions asked and answers given were written down on the spot or directly after the interview. The qualitative data are presented here to provide the contextual information given by the respondents in the survey.

### Data analysis

Information collected in the questionnaires was entered into a database using Epi Info (Epi Info, version 3.5.4). Data was checked for consistency and analysed anonymously. Chi-square test or Fisher’s exact test were used to assess proportions. Continuous variables were converted into ordered categorical variables when suitable. For normally distributed quantitative data, means were compared using Student’s t-test otherwise, the Mann-Whitney U test was used. The Wilcoxon signed rank test was used for comparing related means within individuals when comparing HSB of fever and dengue while pair-wise proportions were compared with a McNemar’s test. Significance was determined at 5% level. Principal components analysis [34, 35] was utilised to weigh socio-economic variables, obtain a relative score and classify individuals into low, average and high socio-economic status. Logistic regression was used to compare crude and adjusted odds ratios (OR). Multivariate logistic regression analysis was used to determine variables independently associated with the intended first action reported in HSB pathways for fever and suspected dengue. Variables with a p-value ≤0.2 after adjusting by age group and sex were fitted into these multivariate models and adjusted for further confounders. Effect modification was analysed and resulting models compared by a likelihood ratio test. Two separate final models are presented, one in the case of fever and the second in the case of intended suspected dengue (Table 1). Data was analysed using SPSS (SPSS Inc., version 20.0, Chicago, Illinois) and STATA (Stata Statistical Software: Release 10. College Station, TX,) softwares.

**Table 1:** Final model of factors independently associated with first intended action in the case of fever and dengue.

Final model of factors independently associated with visiting a doctor as the first intended action versus home treatment in the case of fever and dengue		
	OR (95% CI)	p-value
**FEVER**		
**Education**		
Illiterate/ pre or primary school	1	
Secundary school	0.25 (0.06–1.01)	0.051
University/ university polytechnic	0.18 (0.03–1.05)	0.057
**DENGUE**		
Place of residence		
Candelaria	1	
Cooperativa	1.06 (0.17–6.66)	0.953
Caña de Azúcar	0.28 (0.08–0.93)	0.038
**Children vs. adults**		
Children (caregivers)	1	
Adults	3.33 (1.20–9.21)	0.021
**Reported previous dengue infection**		
No	1	
Yes	0.29 (0.09–0.89)	0.031
**Risk perception**		
Not feeling at risk	1	
Feeling at risk	3.29 (1.04–10.40)	0.042

### Ethic statement

This study was approved by the Ethics Review Committee of the Biomedical Research Institute, Carabobo University (Aval Bioetico #CBIIB(UC)-014), Maracay, Venezuela; the Ethics, Bioethics and Biodiversity Committee (CEBioBio) of the National Foundation for Science, Technology and Innovation (FONACIT) of the Ministry of Science, Technology and Innovation, Caracas, Venezuela; and by the Regional Health authorities of Aragua State (CORPOSALUD Aragua). All adult participants signed written informed consent forms, and a parent or guardian of any child participant provided written informed consent on their behalf. Children between 8 and 17 years old provided written informed assent.

## Results

Between September 2013 and February 2014, we conducted a cross-sectional study within the third annual survey of a dengue community-based cohort study in three neighbourhoods of Maracay city, Venezuela [10]. We aimed to understand the HSB and access to care of a population exposed to hyperendemic dengue transmission. Individual and household-related interviews were conducted targeting adults and parents/guardians of children living in the area of study. Because of violence in the country and in our area of study during anti-governmental protests in February and March 2014 [36–38], the original sample size of 260 subjects was not reached. Overall, 105 individuals were interviewed where 54 interviews referred to HSB of adults (“adult questionnaire”) and 51 to caregivers of children (“child questionnaire”). In addition, 92 household socio-economic questionnaires were applied.

### General characteristics

We described the general features, dengue knowledge and socio-economic characteristics of the study population and compared individuals interviewed with the adult versus the child questionnaire (S1 and S2 Tables). The 105 interviewed individuals had a mean age of 40 years (range: 18–87 years) and were mostly females (86.7%). Children caregivers were older than those queried with the adult questionnaire (mean age 44 vs. 35 years respectively; p<0.001). This was expected, as those who cared for children were mainly mothers or grandmothers. Most of the interviewed individuals lived in Candelaria neighbourhood (68.6%). We were unable to complete the planned interviews in the other two neighbourhoods (Cooperativa and Caña de Azúcar) because of the previously mentioned anti-governmental protests. The majority of the 105 respondents completed secondary school (51.9%) or higher education (31.7%) and were housewives or domestic workers (53.8%). Those interviewed with the adult questionnaire had a higher education level and consisted of a higher proportion of students than parents/guardians of children. As in the rest of the country, the majority of the individuals professed a catholic religion (75.2%) (S1 Table).

Most persons lived in households with five to six rooms (46.7%) and households were mainly occupied by more than five inhabitants (71%). Households of children caregivers were more crowded than those of the ones interviewed with the adult questionnaire (p = 0.003), and had a lower monthly income (p = 0.003) and socio-economic status (p = 0.004). There were no statistically significant differences between adults and parents/guardians with respect to the availability of public services, persons per household and the amount of household rooms (S2 Table).

### Knowledge

The majority of interviewed individuals (n = 103; 98.1%) indicated that they had heard about dengue and showed good knowledge about dengue transmission. In response to the open question “how do you think people get infected by dengue?”, 95.2% (n = 100) mentioned ‘the bite of a mosquito’ or similar. In response to the open question “what are the symptoms of dengue?”, nearly half (n = 47; 44.8%) mentioned up to three correct symptoms, the rest (n = 58; 55.2%) pointed out 4 or more, with a range of 0–8 symptoms. The overall knowledge score ranged from 1–9 correct answers (S1 Table).

### Risk perception and reported previous dengue infection

The majority of the individuals (n = 73; 69.5%) reported to feel at risk of dengue. Almost all subjects (n = 103; 98.1%) also believed that people could die from dengue disease. Feeling at risk was equally reported when referred to children and adults (n = 37; 72.5% vs. n = 36; 69.2%; p = 0.711). Finally, 33 out of 103 respondents (32%) mentioned that they/their child(ren) had dengue previously while one person did not know. Although a previous dengue infection was mentioned more frequently by parents/guardians of children than by adults (36.0% vs. 28.8% respectively) the difference was not significant (p = 0.440).

### Intended health seeking behaviour pathways in the case of fever or suspected dengue

In order to understand the steps people would take in their search for health care, interviewees were confronted with the open questions: ‘what would you do if you/your child had fever’; and ‘what would you do if you think that you/your child had dengue’. In the case of fever, most people chose to first treat fever at home (n = 88; 83.8%) versus only 12 (11.4%) who mentioned that they would first seek medical help. In the case of dengue, the opposite was observed: most people would first visit a doctor (n = 63; 60%), while nearly a third decided they would first treat dengue at home (n = 31; 29.5%; p<0.001). Measuring the temperature at home was mentioned by 35 (33.3%) respondents in the case of fever and 29 (27.6%) in suspected dengue.

Less frequently proposed initial actions in the case of fever were ‘performing blood tests in a laboratory’ (usually referring to a full blood count or platelet count tested at public or private laboratories), ‘inform my/the mother’, and ‘to rest’, while two adults decided they would take no action mentioning as reasons: ‘I will recover myself’, ‘there is no need for visiting a doctor in case of fever’ and ‘I don’t like doctors’. Other first intended actions mentioned with respect to dengue were ‘to perform blood tests’, ‘to call a medical doctor’, ‘to visit an alternative doctor (not further specified)’ and ‘other’ (inform mother, change the clothes of the child and use a mosquito net, call the dengue project staff, evaluate the disease). There were people who mentioned to do a blood test before going to the doctor. A tradeswoman and mother of a 9 years-old child explained this, referring to her daughter (the quote is abbreviated): ‘When I go to the doctor, he will tell me to go to a laboratory to do blood tests. If I do a blood test before going to the doctor, this will save me the cost of one consultation.’ This woman told us she would test for platelets when asked what she would do if she thought her daughter would have dengue.

**Fig 1:**
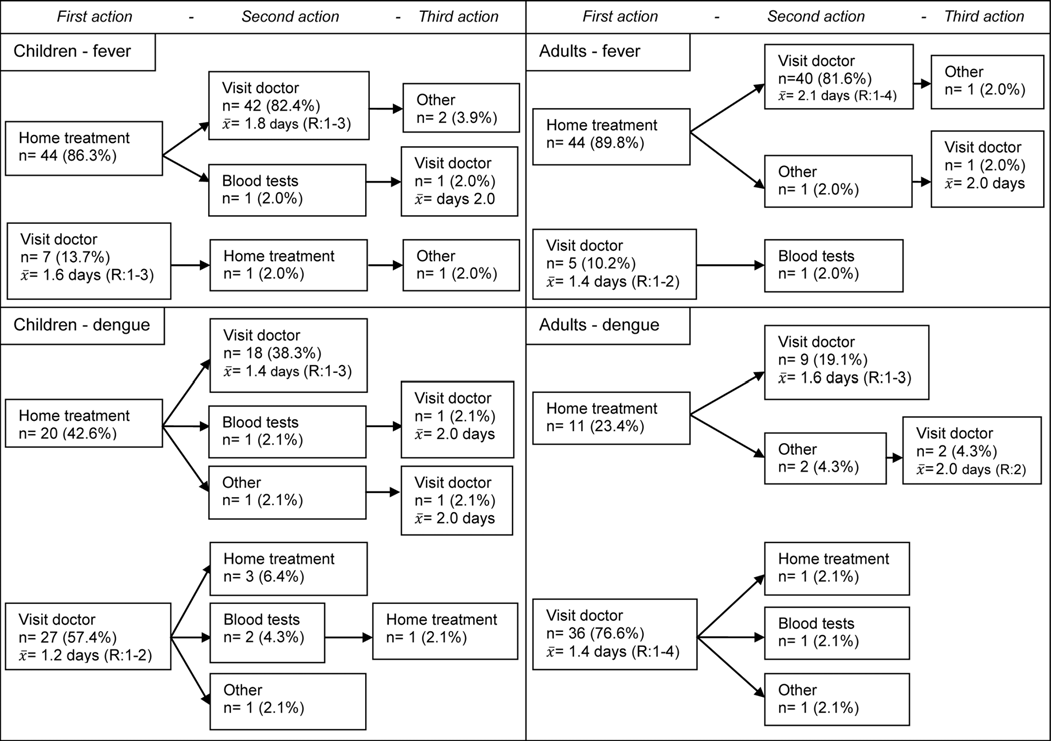
Intended health seeking behaviour pathways in the case of fever and suspected dengue. $\bar{x}$ = mean day when visiting a medical doctor; R = range of day chosen to visit a doctor (min–max). The upper-left and upper-right panel show the first three steps intended to take in the case of fever by parents/guardians referring to their child and adults, respectively. The lower-left and lower-right panel show the first three steps intended to take in the case of suspected dengue by parents/guardians referring to their child and adults, respectively. Percentages correspond with their contribution to the child sample (fever: n = 51 & dengue: n = 47) or the adult sample (fever: n = 49 & dengue: n = 47).

The first three actions individuals anticipated taking in the circumstance of fever or dengue, stratified by behaviour in the case of adults or children are presented as a flowchart in Fig 1. Only the pathways that begin with either ‘home treatment’ or ‘visit medical doctor’ are shown, as these included 92.4% of all pathways. Proportions and analysis from now on in this article referring to Fig 1, are based on the mentioned 92.4% sample.

No differences were found when comparing the intended first actions and pathways between parents/guardians and adults in the case of fever, since most of them decided they would first treat fever at home, as depicted in the upper-left (parents/guardians of children) and upper-right panel (adults) of Fig 1. Concerning dengue, differences were observed when comparing the intended first actions and pathways of children caregivers and adults, as can been seen in the lower left (parents/guardians) and lower right panel (adults) of Fig 1. In the case of dengue, more adults than parents/guardians reported to first visit a doctor (n = 36; 76.6% vs. n = 27; 57.4%; p = 0.048), however children would have been taken earlier to the doctor than adults (mean day chosen to visit a doctor: 1.3 (n = 47) in children versus 1.5 (n = 46) in adults; p = 0.108) (Fig 1). The combination of first treating at home and afterwards visiting a doctor, as a second step in the HSB pathway for suspected dengue, was reported more frequently by caregivers of children compared to adults (n = 18; 38.3% vs. n = 9; 19.1%; p = 0.040)(Fig 1).

### Determinants of intended first action of the HSB pathways for fever or suspected dengue

To explore the association of determinants with the intended first action for both fever and dengue, we compared the characteristics of those who would first treat at home versus those who intended to first visit a doctor in univariate analysis (S3 Table). The tested characteristics included child/adult-sample, age, sex, place of residence, education, occupation, religion, monthly income, socio-economic status, overall knowledge on dengue, reporting a previous dengue infection and risk perception. In the case of fever, those who would first visit a doctor were likely to be people with a lower educational level (p = 0.069), other variables did not show a significant association, probably due to the small sample size of those choosing to visit a doctor (n = 12). In relation to suspected dengue, the participants who chose to treat firstly at home consisted of higher proportions of people living in Caña de Azúcar (p = 0.058) and caregivers of children (p = 0.048). All other tested variables did not show a significant association with any of the intended actions (S3 Table).

Table 1 shows the final multivariate models of factors that remained independently associated with visiting a doctor as the first intended action (instead of choosing home treatment) for either fever or suspected dengue. Respondents with a lower level of education were more likely to seek medical help as their first action in the case of fever, this relation was nearly significant. In the case of suspected dengue, individuals who referred having had a dengue infection in the past preferred to first treat dengue at home instead of going to a doctor firstly. Moreover, feeling at risk of dengue infection, not living in Caña de Azúcar neighbourhood and being an adult (as opposed to a child caregiver) in the case of suspected dengue were directly associated with the intention to first seek medical help (Table 1).

In order to determine whether the intention to first treat at home would make people choose to go later to a doctor, we compared the day of seeking medical care from those who would first treat at home with those who would go firstly to the doctor. Those who intended to first treat at home reported a significant delay in their intentions to seek medical help versus those who intended to go to a doctor in the case of fever (mean day = 1.93 vs. mean day = 1.50; p = 0.039) but not in the case of dengue (mean day = 1.42 vs. mean day = 1.17; p = 0.098).

### Home treatment

**Fig 2:**
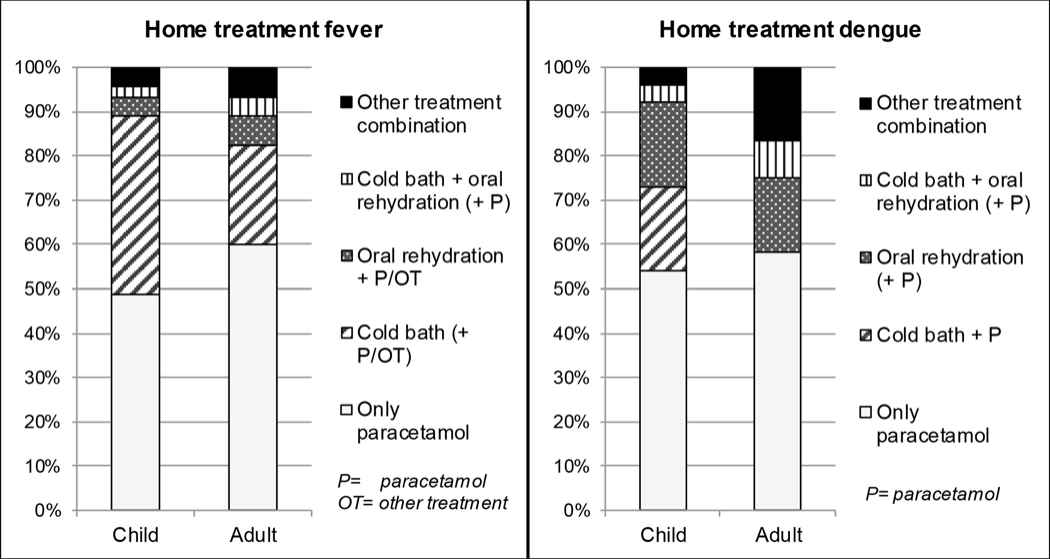
Home treatment choices for fever and dengue. The percentages correspond to their contribution to those who mentioned to treat at home when answering the adult questionnaire (fever: n = 45; dengue: n = 12) or the child questionnaire (fever: n = 45; dengue: n = 26).The categories of treatment combinations in fever and dengue are equal in meaning. However, reporting to treat at home with ‘paracetamol’ (P) and ‘other treatment’ (OT) varies between the categories of home treatment of fever and dengue. P and OT are placed within brackets if not all subjects within the category used additionally P or OT. OT may refer to the following: rubbing the body with alcohol/cream, body sponging, aspirin, other medication or rest.

Overall, 90 (85.7%) individuals stated they would treat fever at home at any time in their health seeking decision process while only 38 (36.2%) would take this decision in the case of suspected dengue infection (p<0.001). Paracetamol (an antipyretic) was the most commonly chosen home treatment overall while taking a cold bath/shower and oral rehydration were the second most common types of home treatments in the case of fever and dengue, respectively (Fig 2).

Within the people who reported to treat fever at home, most would use paracetamol (n = 86, 95.6%) to lower the temperature, followed by a cold bath/shower (n = 31, 34.4%), oral rehydration (n = 8; 8.9%), body sponging with a wet compress or sponge (n = 7; 7.8%) and other ways of home treatment (n = 7; 7.8%) such as rubbing the body with alcohol/cream, taking aspirin, other medication or rest. Paracetamol was also the most common choice among those who would treat dengue at home (n = 30; 78.9%), however, the use of oral rehydration (n = 9; 23.7%) was cited more frequently than in the case of fever, opposite to the use of a cold bath/shower (n = 7; 18.4%). Other (n = 2; 5.3%) home treatments for dengue included body sponging or rest. Combinations of home treatment for fever and dengue stratified by child or adult are shown in Fig 2. The different choices for home treatment showed no significant differences between children and adults.

### Day when seeking medical help

**Fig 3:**
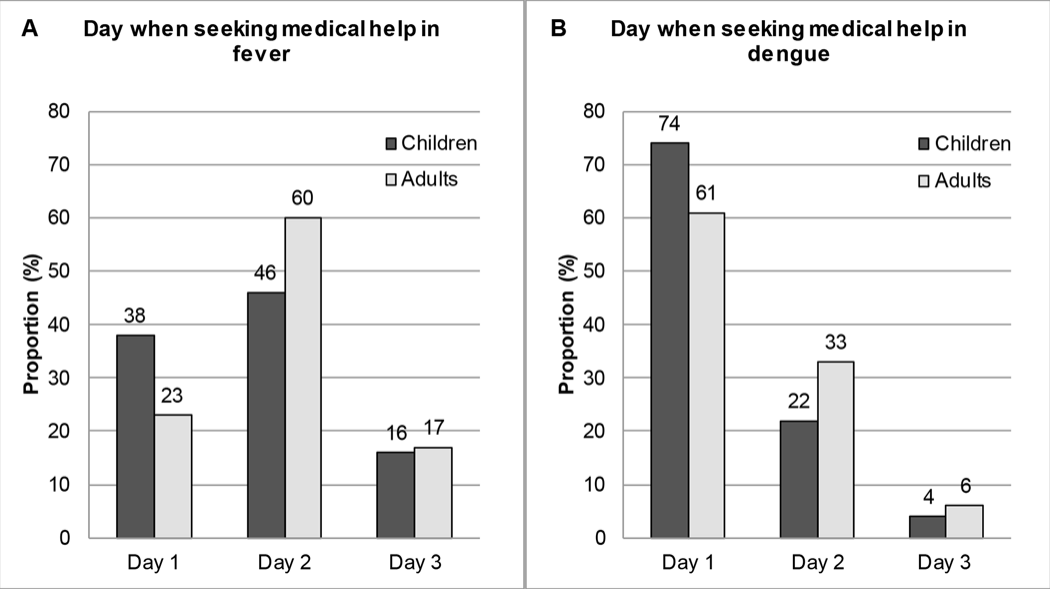
Day when seeking medical help in case of dengue and fever. Section A: children n = 51; adults n = 48. Section B: children n = 51; adults n = 51.

If people reported to seek medical help when they/their child would have fever or suspected dengue, they were subsequently asked on which day after onset of first symptoms they would visit the doctor. Most parents/guardians of children and adults would seek medical help on day 2 after fever onset, but when dengue was suspected most people would go on day one to the doctor (Fig 3). Parents/guardians would take their children earlier to the doctor in case of dengue than in case of fever (mean: 1.30 days vs. 1.78 days; p<0.001). Referred to adults the mean reported day was 1.47 in case of dengue and 1.96 in case of fever (p<0.001). Although children would visit the doctor earlier than adults, this difference was neither significant in the case of fever (p = 0.206) nor for dengue (p = 0.162).

### Reasons for visiting a doctor in the case of suspected dengue

The most frequently reported reasons that prompted individuals to visit a doctor in suspected dengue were the appearance of new symptoms (n = 81; 77.1%), the rise of body temperature (n = 78; 74.3%) and the persistence of fever (n = 35; 33.3%). Almost 10% (n = 10) of the people stated ‘dengue is a severe disease’ as a reason to seek medical help. The most frequent symptoms mentioned were headache (n = 36; 34.3%), corporal pain (n = 32; 30.5%) and weakness (n = 27; 25.7%). Other typical dengue symptoms were mentioned less frequently: rash (n = 18; 17.1%), vomiting (n = 11; 10.5%), eye pain (n = 9; 8.6%) and muscle pain (n = 1; 1.0%). Moreover, warning symptoms were not frequently indicated as reasons to seek medical help: vomiting (n = 11; 10.5%), bleeding (n = 9; 8.6%), abdominal pain (n = 6; 5.7%). The mean temperature referred by interviewees was 39.4°C (range: 38.0°C—42.0°C) while persistence of fever ranged between one to four days with a mean of two days. There were no significant differences when comparing the reasons to seek medical care in suspected dengue between children and adults.

## Discussion

Health seeking behaviour and access to care in relation to dengue disease in the Americas are scarcely described in the literature. Through a cross-sectional household community-based study, we provide insights in the intended HSB and access to care of a population exposed to endemic dengue transmission in Venezuela [10]. We showed that i) there is a difference in intended pathways to care in the case of a suspected dengue infection as opposed to fever, and between children caregivers and adults when dengue is suspected; ii) medical care would be sought earlier in the case of a suspected dengue infection than in the case of fever; and iii) having had a previous dengue infection, being a child carer and living in the neighbourhood Caña de Azúcar were determinants for treating a dengue infection first at home, while feeling at risk of dengue was associated with initial health centre attendance.

Our study found that most individuals intended to look for medical help as their first action if they suspected a dengue infection while treating at home would be their first choice in case of fever only (Fig 1). Considering the HBM, fever is not enough a cue to action to make the respondents proceed as for dengue. The uncertainty of the conditions reduces the ability of fever to increase the perception of susceptibility to dengue [39–44]. Half of the participants in our study identified three or less symptoms characteristic of dengue. Taking into account that a dengue infection is likely to present with fever as a chief symptom, the latter group is likely to initially follow the pathways they described for fever when they actually have dengue. Studies in Colombia [42] and Cambodia [22] found that the majority of dengue infections were first treated at home. These findings correspond with the intended pathway for fever found in the current study, where home treatment was frequently chosen above medical care. Contrariwise, when a dengue infection was suspected, the participants in our study would seek immediate medical attention as was reported in Thailand and Malaysia [25, 26]. Our results show that, if people would identify that they or their children have a dengue infection, they would reach for medical attention earlier and minimise the likelihood of developing complications during dengue disease in agreement with a study in Brazil [45]. These tendencies are encouraging indicating that our study population perceives dengue as a more serious ailment than other causes of fever and is reflected by the reported appearance of new symptoms and a rising body temperature as chief reasons to seek medical help. This is in line with a study in Cambodia, where the perceived severity of dengue illness in children was found to influence the selection of therapeutic options [22].

We show that children’s parents/caretakers and adults have similar HSB intentions when referring to fever, but not when referring to dengue. While in the case of dengue the majority of parents/carers and adults would firstly look for a doctor, a higher proportion of children were initially treated at home (Fig 1, Table 1). However, when comparing the day of seeking medical care of all HSB pathways which included a doctor’s visit, children would still be taken earlier to a doctor than adults (Fig 1). Other studies addressing HSB of dengue only refer either to adults or carers of children without comparisons between these two groups [22, 25, 26, 42]. The results from our study suggest that it may be wise to consider targeting children caregivers and adults separately when health promotion campaigns are performed, since their intended HSB may be different.

Home treatments used in dengue vary according to country or region and include herbs and over-the-counter medicines like paracetamol [22, 46] or liquids such as water [46], carbonated isotonic sports drinks, fruit and vegetable juices and frog or crab soup [26]. We found that fever was treated at home most frequently with paracetamol or a cold/tepid bath, while dengue was mostly managed with paracetamol or oral rehydration. In Venezuela, a cold/tepid bath or showering is indicated by health personnel to rapidly lower the temperature especially in children. Oral rehydration was also frequently mentioned denoting that information obtained at health centres and given during dengue campaigns reaches the population. It is important to stress that from the medical point of view, home treatment of a suspected dengue infection should be followed quickly by a doctor’s visit so that the patient can be properly monitored by medical personnel [1].

Contrary to expected, a previously experienced dengue infection did not necessarily result in an intended prompt health care seeking. This is worrying since those with previous dengue antibodies are more at risk to develop severe disease from a subsequent heterologous dengue infection [47–49]. Efforts should be made to promote prompt doctor attendance for these individuals. Also surprisingly, univariate analysis indicated that a higher education level was associated with the intended choice for home treatment in the case of fever (p = 0.069), in agreement with a study in Thailand [25]. Finally, the place of residence and caring for a child were also associated with intended home treatment of suspected dengue. Caña de Azúcar neighbourhood is one of the most densely populated areas of Maracay [50]. A recent study showed that people in this neighbourhood lived in smaller houses and more crowded conditions (which are surrogate markers of poverty) and were at higher risk of acquiring a recent dengue infection than Candelaria and Cooperativa neighbourhoods [10]. This may explain the lower health centre attendance in agreement with studies on malaria in Africa [51, 52]. We found no other characteristics of the neighbourhood Caña de Azúcar that could explain the preference of its inhabitants to treat a dengue infection firstly at home.

The fact that individuals who felt at risk of dengue were more likely to search firstly medical help has been described earlier [26] and can serve as a focus when promoting timely health centre attendance. Several studies show that (ongoing) reports of outbreaks and media coverage can increase the awareness and risk perception of a disease [53–55]. An increased risk perception may not only encourage immediate health centre attendance, but also influences community participation in protective behaviour [15, 26, 54]. Therefore, timely, complete and trustworthy information on the dengue and health situation in a country should positively impact the population at risk with regards to early health centre attendance and improvement in protective behaviour. These messages should 1) promote prompt HC attendance when dengue is suspected (“visit your doctor as soon as you think you have dengue”), and 2) stress the importance of attention to warning symptoms of dengue when fever recedes (“when fever goes, look for the following warning signs in yourself or your sick child: severe abdominal pain, persistent vomiting, etc”).

### Limitations

Due to the political unrest that took place during the study period, data collection was not completed for all three neighbourhoods and the majority of the interviewees resided in Candelaria neighbourhood. However, this population was considered to be representative of most urban areas from Maracay city minimising selection bias. Education, monthly income and socio-economic status were significantly lower in the child sample, nonetheless these variables were controlled for in the multivariate analysis. The difference in socio-demographic and socio-economic characteristics between the adults and parents/guardians of children can be attributed to the fact that almost half of the children in Aragua State, Venezuela, are part of a household run by a single mother, who have generally a lower degree of education and a lower income [30].

A strength of the study was that data was collected from a well characterised cohort study population of which socio-economic and epidemiological data was available. Moreover, contrary to hospital-based studies, our study design made us able to include people who would avoid attending health centres, thus obtaining insight in their intended HSB. Furthermore, people were interviewed at home, providing a safe and confident environment. Finally, the analysis was strengthened with the conceptualisation that included aspects of the HBM. For future studies, we recommend to apply the conceptualisation at the stage of data collection.

### Conclusions

In the current study, we were able to describe intended HSB in the case of dengue and fever at community level. Our results suggest that for the people who intend to seek medical care in the case of a dengue infection, self-diagnosis might be an obstacle. The differences found in HSB between fever and dengue imply that fever does not increase the perceptions of susceptibility to dengue. As mentioned before, a delay in care-seeking is associated with a higher mortality and complications during dengue disease [1, 12]. Therefore, the early intended medical care-seeking in the case of a suspected dengue infection shown in this study suggests a possible improvement of HSB and prognosis if an algorithm or tool can be designed to diagnose dengue at home. In addition, we found that those who previously had a dengue infection were more likely to treat a next infection firstly at home. Since this group has a higher chance of developing a severe dengue disease, efforts have to be made to promote prompt health centre attendance in this group. In this, raising awareness and risk perception of dengue by media coverage and information at health centres may improve this favourable behaviour. Comparing the results of the current study (HSB intentions) with those of the actual HSB taken by people, such as in health centre-based studies, could reveal other possible barriers for achieving the intended HSB. More community and health centre-based studies should be performed to achieve a wider view and stronger conclusions on HSB for people exposed to dengue in the Americas.

## Supporting Information

S1 Table Socio-demographic characteristics and knowledge about dengue of interviewed individuals. ^a^ p-value corresponds to the comparison between the child and adult questionnaire responses; ^b^From the total sample, only one person was illiterate; ^c^From the total sample, one person was a Jehovah’s witness; ^d^Range overall knowledge score: 1–9 correct answers; *Fishers exact test.
(PDF)

S2 Table Socio-economic characteristics of interviewed individuals. ^a^ p-value corresponds to the comparison between the child and adult questionnaire responses; ^b^Number of rooms, bathrooms not included; ^c^ Minimum wages at time of the study was 2703 VEF—3270 VEF; *Fishers exact test.
(PDF)

S3 Table Socio-demographic and socio-economic characteristics related to the intended first action of the HSB pathway in the case of fever and dengue; home treatment vs. visiting a doctor. ^a^n = 86 for subjects choosing home treatment in case of fever; n = 30 for subjects choosing home treatment in case of dengue; n = 62 for subjects choosing visiting a doctor in case of dengue; ^b^ p-value corresponds to the comparison between intending to treat at home or visiting a doctor as first action for each case: fever or suspected dengue. ^c^n = 87 for subjects choosing home treatment in case of fever; n = 62 for subjects choosing visiting a doctor in case of dengue; ^d^From the total sample, there was one person illiterate; ^e^n = 85 for subjects choosing home treatment in case of fever; n = 11 for subjects choosing visiting a doctor in case of fever; n = 30 for subjects choosing home treatment in case of dengue; n = 61 for subjects choosing visiting a doctor in case of dengue;^f^From the total sample, one person was a Jehovah’s witness; ^g^n = 64 for subjects choosing home treatment in case of fever; n = 8 for subjects choosing visiting a doctor in case of fever; n = 25 for subjects choosing home treatment in case of dengue; n = 45 for subjects choosing visiting a doctor in case of dengue; ^h^n = 76 for subjects choosing home treatment in case of fever; n = 10 for subjects choosing visiting a doctor in case of fever; n = 27 for subjects choosing home treatment in case of dengue; n = 54 for subjects choosing visiting a doctor in case of dengue; ^i^n = 86 for subjects choosing home treatment in case of fever; n = 30 for subjects choosing home treatment in case of dengue; ^j^n = 87 for subjects choosing home treatment in case of fever; n = 30 for subjects choosing home treatment in case of dengue; *Fisher’s exact test.
(PDF)
