# Correction: Health Seeking Behaviour and Treatment Intentions of Dengue and Fever: A Household Survey of Children and Adults in Venezuela

**DOI:** 10.1371/journal.pntd.0004366

**Published:** 2016-01-04

**Authors:** The PLOS Neglected Tropical Diseases Staff

S3 Table is a duplicate of S2 Table. Please view the correct S3 Table below.

## Supporting Information

S3 Table Supplementary dataset. This file includes supplementary data.
(PDF)
