# Supplementary material for: Health Seeking Behaviour and Treatment Intentions of Dengue and Fever: A Household Survey of Children and Adults in Venezuela
Source: PLoS Negl Trop Dis. 2015 Dec 1;9(12):e0004237. doi: 10.1371/journal.pntd.0004237 (PMC4666462; doi:10.1371/journal.pntd.0004237)
Supplement: S1 Table — a p-value corresponds to the comparison between the child and adult questionnaire responses; bFrom the total sample, only one person was illiterate; cFrom the total sample, one person was a Jehovah’s witness; dRange overall knowledge score: 1–9 correct answers; *Fishers exact test. (PDF) [file pntd.0004237.s001.pdf]

S1 Table. Socio-demographic characteristics and knowledge about dengue of interviewed individuals.

	Total (n=105)		Child questionnaire (n=51)		Adult questionnaire (n=54)		
**Age** (n=103)	n	(%)	n	(%)	n	(%)	p-value[a]
18-30	39	(37.9)	8	(16.3)	31	(57.4)	
31-50	34	(33.0)	25	(51.0)	9	(16.7)	
>50	30	(29.1)	16	(32.7)	14	(25.9)	<0.001
**Sex** (n=105)							
Females	91	(86.7)	47	(92.1)	44	(81.5)	
Males	14	(13.3)	4	(7.8)	10	(18.5)	0.108
**Place of residence** (n=105)							
Candelaria	72	(68.6)	36	(70.6)	36	(66.7)	
Cooperativa	11	(10.5)	4	(7.8)	7	(13.0)	
Caña de Azúcar	22	(21.0)	11	(21.6)	11	(20.4)	0.693
**Education** (n=104)							
Illiterate/ pre or primary school[b]	17	(16.3)	11	(21.6)	6	(11.3)	
Secondary school	54	(51.9)	31	(60.8)	23	(43.4)	
University/ university polytechnic	33	(31.7)	9	(17.6)	24	(45.3)	0.009
**Occupation** (n=104)							
Student	16	(15.4)	4	(7.8)	12	(22.6)	
Housewife/Domestic-/manual worker	56	(53.8)	34	(66.7)	22	(41.5)	
Merchant/ Employee/ Office worker/ Professional/ University staff	32	(30.8)	13	(25.5)	19	(35.8)	0.022
**Religion** (n=101)							
No religion	6	(5.9)	0	(0.0)	6	(11.3)	
Catholic	76	(75.2)	37	(77.1)	39	(73.6)	
Christian/Protestant/Evangelist[c]	19	(18.8)	11	(22.9)	8	(15.1)	0.046*
**Overall knowledge score on dengue**[d] (n=105)							
≤4 correct answers	48	(45.7)	21	(41.2)	27	(50.0)	
≥5 correct answers	57	(54.3)	30	(58.8)	27	(50.0)	0.364

Legend S1 Table: [a] p-value corresponds to the comparison between the child and adult questionnaire responses; [b]From the total sample, only one person was illiterate; [c]From the total sample, one person was a Jehovah's witness; [d]Range overall knowledge score: 1-9 correct answers; *Fishers exact test.
