# Supplementary material for: Health Seeking Behaviour and Treatment Intentions of Dengue and Fever: A Household Survey of Children and Adults in Venezuela
Source: PLoS Negl Trop Dis. 2015 Dec 1;9(12):e0004237. doi: 10.1371/journal.pntd.0004237 (PMC4666462; doi:10.1371/journal.pntd.0004237)
Supplement: S2 Table — a p-value corresponds to the comparison between the child and adult questionnaire responses; bNumber of rooms, bathrooms not included; c Minimum wages at time of the study was 2703 VEF—3270 VEF; *Fishers exact test. (PDF) [file pntd.0004237.s002.pdf]

S2 Table. Socio-economic characteristics of interviewed individuals.

	Total (n=105)		Child quest. (n=51)		Adult quest. (n=54)		
**Availability of complete public services** (n=91)	n	(%)	n	(%)	n	(%)	p-value[a]
No	4	(4.4)	3	(6.8)	1	(2.1)	
Yes	87	(95.6)	41	(93.2)	46	(97.9)	0.284*
**Persons per household** (n=87)							
2-4	25	(28.7)	11	(27.5)	14	(29.8)	
5-6	28	(32.2)	12	(30.0)	16	(34.0)	
≥7	34	(39.1)	17	(42.5)	17	(36.2)	0.831
**Household rooms**[b] (n=90)							
2-4	35	(38.9)	21	(47.7)	14	(30.4)	
5-6	42	(46.7)	18	(40.9)	24	(52.2)	
≥7	13	(14.4)	5	(11.4)	8	(17.4)	0.234
**Crowding (persons/room)** (n=86)							
<1.50	57	(66.3)	20	(50.0)	37	(80.4)	
≥1.50	29	(33.7)	20	(50.0)	9	(19.6)	0.003
**Monthly income (VEB)**[c] (n=76)							
≤7000 VEB (minimum wages)	38	(50.0)	26	(66.7)	12	(32.4)	
>7000 VEB	38	(50.0)	13	(33.3)	25	(67.6)	0.003
**Socio-economic status** (n=90)							
Low	38	(42.2)	23	(52.3)	15	(32.6)	
Average	30	(33.3)	17	(38.6)	13	(28.3)	
High	22	(24.4)	4	(9.1)	18	(39.1)	0.004

Legend S2 Table: [a] p-value corresponds to the comparison between the child and adult questionnaire responses; [b]Number of rooms, bathrooms not included; [c] Minimum wages at time of the study was 2703 VEF - 3270 VEF; *Fishers exact test.
