# Supplementary material for: Correction: Health Seeking Behaviour and Treatment Intentions of Dengue and Fever: A Household Survey of Children and Adults in Venezuela
Source: PLoS Negl Trop Dis. 2016 Jan 4;10(1):e0004366. doi: 10.1371/journal.pntd.0004366 (PMC4699773; doi:10.1371/journal.pntd.0004366)
Supplement: S3 Table — This file includes supplementary data. (PDF) [file pntd.0004366.s001.pdf]

Table S3. Socio-demographic and socio-economic characteristics related to the intended first action of the HSB pathway in the case of fever and dengue; home treatment vs. visiting a doctor.

	Fever				Dengue			
	Home treatment n=88		Visit a doctor n=12		Home treatment n=31		Visit a doctor n=63	
**Age**[a]	n	(%)	n	(%)	n	(%)	n	(%)
18-30	32	(37.2)	2	(16.7)	10	(33.3)	21	(33.9)
31-50	30	(34.9)	4	(33.3)	13	(43.3)	20	(32.3)
>50	24	(27.9)	6	(50.0)	7	(23.3)	21	(33.9)
p-value[b]	0.268*				0.489			
**Sex**								
Females	78	(88.6)	11	(91.7)	29	(93.5)	53	(84.1)
Males	10	(11.4)	1	(8.3)	2	(6.5)	10	(15.9)
p-value[b]	1.000*				0.325*			
**Place of residence**								
Candelaria	61	(69.3)	8	(66.7)	17	(54.8)	48	(76.2)
Cooperativa	9	(10.2)	2	(16.7)	3	(9.7)	6	(9.5)
Caña de Azúcar	18	(20.5)	2	(16.7)	11	(35.5)	9	(14.3)
p-value[b]	0.787*				0.058*			
**Education**[c]								
Illiterate/ pre or primary school[d]	12	(13.8)	5	(41.7)	3	(9.7)	14	(22.6)
Secondary school	48	(55.2)	5	(41.7)	18	(58.1)	30	(48.4)
University/ university polytechnic	27	(31.0)	2	(16.7)	10	(32.3)	18	(29.0)
p-value[b]	0.069*				0.312			
**Occupation**[c]								
Student	13	(14.9)	2	(16.7)	7	(22.6)	7	(11.3)
Housewife/Domestic-/manual worker	48	(55.2)	8	(66.7)	18	(58.1)	35	(56.6)
Merchant/ Employee/ Office worker/ Professional/ University staff	26	(29.9)	2	(16.7)	6	(19.4)	20	(32.3)
p-value[b]	0.686*				0.231*			
**Religion**[e]								
No religion	3	(3.5)	1	(9.1)	1	(3.3)	3	(4.9)
Catholic	67	(78.8)	7	(63.6)	21	(70.0)	50	(82.0)
Christian/Protestant/Evangelist[f]	15	(17.6)	3	(27.3)	8	(26.7)	8	(13.1)
p-value[b]	0.327*				0.250*			
**Monthly income (VEB)**[g]								
≤7000 VEB	32	50.0	5	62.5	14	56.0	22	48.9
>7000VEB	32	50.0	3	48.6	11	44.0	23	51.1
p-value[b]	0.711*				0.568			
**Socio-economic status**[h]								
Low	33	(43.4)	5	(50.0)	10	(37.0)	25	(46.3)
Average	27	(35.5)	3	(30.0)	11	(40.7)	17	(31.5)
High	16	(21.1)	2	(20.0)	6	(22.2)	12	(22.2)
p-value[b]	1.000*				0.669			
**Child/adult – sample**								
Child	44	(50.0)	7	(51.0)	20	(64.5)	27	(42.9)
Adult	44	(50.0)	5	(59.0)	11	(35.5)	36	(57.1)
p-value[b]	0.588				0.048			
**Overall knowledge dengue**								
≤4 correct answers	38	(43.2)	7	(58.3)	15	(48.4)	28	(45.7)
≥5 correct answers	50	(56.8)	5	(41.7)	16	(51.6)	35	(54.3)
p-value[b]	0.322				0.829			
**Reported previous dengue infection**[i]								
No	58	(67.4)	10	(83.3)	18	(60.0)	46	(73.0)
Yes	28	(32.6)	2	(16.7)	12	(40.0)	17	(27.0)
p-value[b]	0.334*				0.205			
**Risk perception**[j]								
Not feeling at risk	24	(27.6)	3	(25.0)	11	(36.7)	14	(22.2)
Feeling at risk	63	(72.4)	9	(75.0)	19	(63.3)	49	(77.8)
p-value[b]	1.000*				0.142			

Legend S3 Table: [a]n=86 for subjects choosing home treatment in case of fever; n=30 for subjects choosing home treatment in case of dengue; n=62 for subjects choosing visiting a doctor in case of dengue; [b] p-value corresponds to the comparison between intending to treat at home or visiting a doctor as first action for each case: fever or suspected dengue. [c]n=87 for subjects choosing home treatment in case of fever; n=62 for subjects choosing visiting a doctor in case of dengue; [d]From the total sample, there was one person illiterate; [e]n=85 for subjects choosing home treatment in case of fever; n=11 for subjects choosing visiting a doctor in case of fever; n=30 for subjects choosing home treatment in case of dengue; n=61 for subjects choosing visiting a doctor in case of dengue;[f]From the total sample, one person was a Jehovah's witness; [g]n=64 for subjects choosing home treatment in case of fever; n=8 for subjects choosing visiting a doctor in case of fever; n=25 for subjects choosing home treatment in case of dengue; n=45 for subjects choosing visiting a doctor in case of dengue; [h]n=76 for subjects choosing home treatment in case of fever; n=10 for subjects choosing visiting a doctor in case of fever; n=27 for subjects choosing home treatment in case of dengue; n=54 for subjects choosing visiting a doctor in case of dengue; [i]n=86 for subjects choosing home treatment in case of fever; n=30 for subjects choosing home treatment in case of dengue; [j]n=87 for subjects choosing home treatment in case of fever; n=30 for subjects choosing home treatment in case of dengue; *Fisher's exact test.
